# Environmentally Sustainable Food Consumption: A Review and Research Agenda From a Goal-Directed Perspective

**DOI:** 10.3389/fpsyg.2020.01603

**Published:** 2020-07-10

**Authors:** Iris Vermeir, Bert Weijters, Jan De Houwer, Maggie Geuens, Hendrik Slabbinck, Adriaan Spruyt, Anneleen Van Kerckhove, Wendy Van Lippevelde, Hans De Steur, Wim Verbeke

**Affiliations:** ^1^BE4LIFE, Department of Economics and Business Administration, Ghent University, Ghent, Belgium; ^2^BE4LIFE, Department of Psychology and Educational Sciences, Ghent University, Ghent, Belgium; ^3^BE4LIFE, Department of Agricultural Economics, Ghent University, Ghent, Belgium

**Keywords:** environmental sustainable consumption, environmental sustainable food, goal-directed, positive value, perceived discrepancy, behavioral intention, goal intention, act

## Abstract

The challenge of convincing people to change their eating habits toward more environmentally sustainable food consumption (ESFC) patterns is becoming increasingly pressing. Food preferences, choices and eating habits are notoriously hard to change as they are a central aspect of people’s lifestyles and their socio-cultural environment. Many people already hold positive attitudes toward sustainable food, but the notable gap between favorable attitudes and actual purchase and consumption of more sustainable food products remains to be bridged. The current work aims to (1) present a comprehensive theoretical framework for future research on ESFC, and (2) highlight behavioral solutions for environmental challenges in the food domain from an interdisciplinary perspective. First, starting from the premise that food consumption is deliberately or unintentionally directed at attaining goals, a goal-directed framework for understanding and influencing ESFC is built. To engage in goal-directed behavior, people typically go through a series of sequential steps. The proposed theoretical framework makes explicit the sequential steps or hurdles that need to be taken for consumers to engage in ESFC. Consumers need to positively value the environment, discern a discrepancy between the desired versus the actual state of the environment, opt for action to reduce the experienced discrepancy, intend to engage in behavior that is expected to bring them closer to the desired end state, and act in accordance with their intention. Second, a critical review of the literature on mechanisms that underlie and explain ESFC (or the lack thereof) in high-income countries is presented and integrated into the goal-directed framework. This contribution thus combines a top-down conceptualization with a bottom-up literature review; it identifies and discusses factors that might hold people back from ESFC and interventions that might promote ESFC; and it reveals knowledge gaps as well as insights on how to encourage both short- and long-term ESFC by confronting extant literature with the theoretical framework. Altogether, the analysis yields a set of 33 future research questions in the interdisciplinary food domain that deserve to be addressed with the aim of fostering ESFC in the short and long term.

## Introduction

Climate change endangers unique eco-systems, leads to more extreme weather events, reduces biodiversity, and in many ways threatens our current way of living (O’Neill et al., 2017). Household food consumption gives rise to more than 60% of global Greenhouse Gas emissions and between 50 and 80% of total resource use (Ivanova et al., 2016). Thus, making people’s eating patterns more environmentally sustainable is becoming ever more important (Springmann et al., 2016; Hartmann and Siegrist, 2017; Magrini et al., 2018; Hedin et al., 2019). Particularly in high-income countries, transforming food consumption is deemed an essential condition for reaching global sustainability goals (UN, 2016). The current review therefore focuses on different behavioral strategies to promote environmentally sustainable food consumption in high-income countries.

Environmentally Sustainable Food Consumption (ESFC) can be defined as the use of food products “that respond to basic needs and bring a better quality of life, while minimizing the use of natural resources, toxic materials and emissions of waste and pollutants over the life cycle, so as not to jeopardize the needs of future generations” (Oslo Roundtable on Sustainable Production and Consumption, 1994). Major examples of ESFC include increasing consumption of plant-based (Lea et al., 2006) or insect-based foods (Megido et al., 2016), while decreasing meat consumption (Hoek et al., 2004), and opting for seasonal products (Macdiarmid, 2014). In some but not all instances, buying locally produced (MacGregor and Vorley, 2006) and/or organically produced food (Hughner et al., 2007) may also be more environmentally sustainable.

Food preferences, choices and habits occupy a central role in human cultures and food consumption goes far beyond its functional role as a means to survive. Food habits are notoriously hard to change as they are a central aspect of people’s lifestyles (Sonestedt et al., 2005; Flaherty et al., 2018) and their socio-cultural environment (Wright et al., 2001; Carrus et al., 2018; Cairns, 2019). Food choices are also subject to marketing efforts of food companies that have caused changes in dietary norms, in food and drink category preferences (at population level) and in the cultural values underpinning food behaviors (Cairns, 2019). The complexity of food related decisions makes them susceptible to a wide range of social, cognitive, affective, and environmental influences (Bublitz et al., 2010). In sum, efforts to promote ESFC compete with other contextual influences on people’s food choices.

Against this backdrop, it is hardly surprising that many consumers express environmental concern but do not consistently act on it. That is, consumer attitudes toward environmental sustainability are mainly positive, but there is a notable gap between favorable attitudes and actual purchase of sustainable food products, i.e., the attitude-behavior gap (Vermeir and Verbeke, 2006; van Dam and van Trijp, 2013; Aschemann-Witzel and Zielke, 2017).

By formulating a comprehensive theoretical framework in which we integrate academic insights and research findings from different disciplines, the current work aims to contribute to behavioral solutions for environmental challenges in the food domain. First, a goal-directed framework for understanding and influencing ESFC is built. The core assumption of the framework is that, like most human behavior, food consumption is either deliberately or unintentionally directed at attaining goals (Otto et al., 2014). From a goal-directed perspective, food consumption can be directed at the goal of minimizing adverse environmental impact, but people also buy and eat food products to satisfy hunger, to achieve sensory pleasure, to signal social status, to comply with norms and reference groups, etc.

Secondly, we critically reviewed the literature on explanatory, underlying mechanisms related to ESFC in high-income countries, and integrated extant research insights in our framework. Our intended contribution is to answer two key questions: What factors prevent or favor ESFC? And what are the most effective strategies to promote ESFC? While our primary focus is on ESFC, we also include research insights on sustainable consumption in general to highlight potential avenues for future research in the domain of ESFC. By confronting the extant literature with the goal-directed framework, we aim to reveal knowledge gaps as well as insights into how to encourage both short- and long-term ESFC.

The current review differs from previous reviews in several respects. First, we combine a top-down conceptualization with a bottom-up literature review. That is, we start from a comprehensive theoretical framework of goal-directed behavior that delineates necessary components that must be in place for ESFC to occur. Next, we evaluate the extant research (based on a structured, narrative literature review) and identify research gaps based on the framework. Most other reviews build their frameworks only on the basis of reviewed studies, or build their frameworks on the basis of commonly applied theories e.g., Theory of Planned Behavior or Value theory (Aertsens et al., 2009), or limited their literature review to these theoretical applications (Bamberg and Möser, 2007), resulting in a kind of research myopia. That is, by putting too much focus on what is already done and known, there is a risk of missing opportunities and shortcomings that have not yet been studied. Because our framework is constructed independently from the screening of the literature, our framework is well positioned to uncover gaps in research (and thus, to help prioritize future research). This approach can also be used to take stock of the literature on a regular basis, which is essential as the literature on ESFC is growing at an exponential rate (Popescu et al., 2019).

Second, our focus is on identifying research gaps on ESFC. On the one hand, this makes our scope more specific than other reviews concerned with sustainable consumption in general (e.g., White et al., 2019). This allows us to (also, but not only) zoom in on research that may not generalize beyond the context of ESFC. On the other hand, our review is less narrow in scope than other recent reviews that focus, for example, on the transition from meat to plant-based diets (Graca et al., 2019) or on social desirability bias in ecological food research (Cerri et al., 2019). This makes the current review relevant to a broader audience interested in the current state-of-the-art concerning ESFC. Note that we do not study food production, processing, packaging, storage or food waste, for which research/interventions on whole ecosystems are also urgently needed.

Third, many researchers focus either on identifying the “green consumer” segment (Verain et al., 2012) or on specific drivers and barriers of ESFC and their boundary conditions (White et al., 2019). We start from the behavioral process itself by looking into the steps people go through when engaging in goal-directed behavior. We identify for each step interventions that can support people in taking these steps. In doing so, we go beyond work on the predictors of sustainable consumption or behavioral intention (for reviews, see for example Milfont and Markowitz, 2016; Rana and Paul, 2017; White et al., 2019) by suggesting a behavioral process-driven framework that shows how the environment can be influenced via effective interventions as a means to realize enduring behavioral change in ESFC. Hereby we offer both researchers and practitioners a guidance for further research. Notwithstanding the relevance of changes at the macro-level and meso-level (including legislation, taxation, infrastructure, etc., Prothero et al., 2011; Garnett et al., 2015; Reisch and Thøgersen, 2015), the focus of the current review is on micro-level interventions.

## A Goal-Directed Framework Applied to ESFC and Interventions to Promote ESFC

Goals can be defined as desired end states. Hence, goal-directed behavior can be defined as behavior directed at attaining a desired end state (Kruglanski et al., 2015). These definitions imply that for goal-directed behavior to occur, several components need to be in place. Following the work done by Moors et al. (2017) and Moors (2019), we propose a model that posits five components: consumers need to: (1) positively value the environment, (2) discern a discrepancy between the desired versus the actual state of the environment, (3) opt for action to reduce the discrepancy (i.e., goal intention), (4) intend to engage in behavior that is expected to bring them closer to the desired end state (i.e., behavioral intention), and (5) act in accordance with their intention.

First, the end state at which a behavior is directed needs to have a positive value. If an end state is not valued, it will not be pursued. For instance, you are unlikely to reduce your red meat consumption for ecological reasons if you do not want to reduce your ecological footprint. Second, people will engage in goal-directed behavior only when they perceive a discrepancy between the current state and the end state that they value. If there is no perceived discrepancy, there is no reason to act with the aim of reducing the discrepancy. For instance, if you value a low ecological footprint, you are less likely to reduce your red meat consumption to lower your footprint if you think that your ecological footprint is already low. Third, even when there is a perceived discrepancy between the current state and a desired end state, people might choose not to act to accomplish their goal but rather to subjectively devalue the desired end state (so that it is no longer important to pursue this state) or to change their beliefs about the discrepancy between the current and desired state (so that it is no longer necessary to act in order to reduce the discrepancy). For instance, when you know that your ecological footprint is high and you want to lower it by reducing red meat consumption, you might decide that lowering your ecological footprint is not that important in the short term anyway or you might compare your own ecological footprint to people who perform even worse than you, concluding your footprint is actually okay. Fourth, when people do decide to act in order to reduce a perceived discrepancy between the current state and a desired end state, they still need to decide how to act. It is typically assumed that they will choose an action of which they expect that it will bring them closer to the desired end state (e.g., Kruglanski et al., 2015). For instance, people are more likely to lower their red meat consumption than to lower soft drink intake if they expect that lowering red meat consumption is more likely to reduce their ecological footprint than lowering their soft drink intake. Once they have selected a behavior that is expected to bring them closer to the desired end state, we can say that they have formed a behavioral intention, that is, the goal to engage in a behavior that is expected to bring about a desired end state. Fifth, not all behavioral intentions are realized. A first class of hurdles for action relate to the ability of the individual to perform a behavior. If it is impossible to perform the intended behavior, it will not take place. For instance, someone is unlikely to switch to a vegetables-only diet if he/she does not have a clue where to buy such food or how to prepare it. A second reason for not performing an intended behavior relates to other goals that the individual strives for. For instance, it could be that the intended action not only promotes the goal at which it is directed but also hinders the attainment of other goals. If the benefits in terms of one goal are smaller than the costs in terms of other goals, then the intended behavior will not be executed. For instance, you are less likely to lower red meat consumption if you believe that you need the proteins from red meat to strengthen your muscles.

Although many models of goal-directed behavior have been put forward in the literature (e.g., Carver and Scheier, 1981; Locke and Latham, 1990; Bagozzi, 1992; Bagozzi and Dholakia, 1999; Gollwitzer, 1999), we focused on the ideas proposed by Moors et al. (2017) because they provide a uniquely detailed overview of the specific components of goal-directed behavior, that is, the various decision steps that people go through, starting from when they set their goal until they accomplish it. Moreover, we followed the extension of this framework by Moors and colleagues (Moors, 2019; Köster et al., 2020) in which they provided a systematic overview of the types of problems that can arise in each of the steps of the decision process as well as types of solutions. The specific contribution of the current paper lies in (a) the application of this extended framework to the domain of ESFC as a tool for organizing the literature and (b) highlighting behavioral solutions to promote ESFC. As such, we organize the literature in terms of the different steps put forward by Moors and colleagues and extend previous literature by identifying interventions that can help people to take these steps to accomplish ESFC. When, in the remainder of this paper, we refer to “our framework” or “our conceptual model,” we thus refer to the extended framework of Moors (2019) and Köster et al. (2020) as it is applied to ESFC.

**FIGURE 1:**
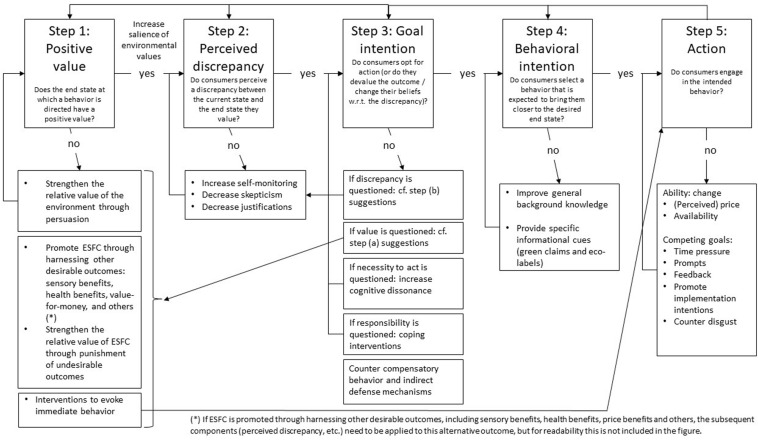
A Goal-Directed Framework Applied to ESFC.

For each component of our framework, we highlight which interventions could take place so that all conditions are met for individuals to engage in ESFC. Figure 1 shows an overview of our framework and the related interventions. An overview of the suggested future research questions can be found in Table 1.

**TABLE 1:** Overview of future research questions.

Positive value	(1) To what extent does activating personal norms strengthen environmental values? (2) Which verbalizations and visualizing techniques increase the value of the environment? (3) When does positive cueing increase the value of the environment? (4) To what extent and when do interventions activating social norms affect the value of the environment? (5) To what extent and under what conditions can fear appeals enhance value of the environment? (6) What is the interplay between interventions strengthening the relative value of the environment and how do they call upon people with either negative/absent/latent/salient pro-environmental values? (7) To what extent, when and why does stressing sensory aspects or using anthropomorphic techniques increase ESFC? (8) Under which conditions can interventions stressing health benefits increase ESFC? (9) To what extent can ESFC be increased by stressing value for money? (10) Which non pro-environmental (perceived) benefits can act as potential reasons for engaging in ESFC? (11) To what extent can ESFC be increased by providing (non-) financial incentives? (12) Which conditions or interventions can create solid consumer support for taxes on non-ecological alternatives? (13) Which nudging interventions positively affect ESFC; to what extent do nudging interventions influence ESC by increasing public awareness or environmental values?
Perceived discrepancy	(14) Which interventions decrease skepticism toward environmental issues? (15) To what extent and how can justifications be minimized?
Goal intention	(16) To what extent can increasing cognitive dissonance increase goal intention? (17) To what extent can evoking guilt or pride or stressing coping mechanisms increase an individual’s sense of personal responsibility and goal intention? (18) How can compensatory beliefs, licensing and the negative footprint illusion be countered? (19) Which indirect defense mechanisms do people use and how can they be reduced?
Behavioral intention	(20) Which typology of labels can bring structure to the labeling literature? (21) What is the moderating effect of labeling characteristics on their effectiveness? (22) How to effectively communicate (multiple) environmentally relevant product attributes (other than organic)? (23) How do different eco-labels interact and how do eco-labels interact with other types of labels and other information?
Action	(24) Which interventions decrease prices and price perceptions? (25) Which interventions are effective for less affluent target groups? (26) How can digital displays, mobile apps, gamification and social media trigger ESFC? (27) How can implementation intentions increase the probability that behavioral intentions are translated in actual ESFC actions? (28) Which behavioral interventions can counter disgust reactions to environmental sustainable foods that are perceived as (visually) unappealing?
General directions for future research	(29) Investigate whether combining different interventions aimed at enhancing ESFC produces add-on effects. (30) Test the long term effects of interventions and how interventions should be adapted to have long-term effects. (31) How to assess sustainability of a food product and how to clearly communicate this environmental impact to customers? (32) Do implicit attitudes predict other sustainable behaviors than explicit attitudes and how can both types of attitudes be changed using the same or different interventions? (33) How to measure attitudes that more closely align to the more concrete level at which actual food choices are being made by consumers in their daily lives?

We illustrate these interventions with several examples from previous research within the domains of behavioral economics, social and personality psychology, communication and behavioral sciences, and food and agricultural economics. To select the literature, we conducted a structured literature search in Web of Science combining specific keywords indicating the environmental friendly character (e.g., “environmental sustainable,” “ecological”) with the consumption aspect (e.g., “consumption,” “choice”) and the food aspect (e.g., “food,” “eating”). This resulted in 60268 papers. We refined our search to include food sciences, behavioral sciences, business, psychology, economics, and management journals and papers published between 2010 and 2020. Within the frame of selected papers published between 2010 and 2015, we only selected those that were cited three or more times (indicating the paper’s relevance) resulting in 3648 papers. These papers were screened on quality and relevance which were determined through consensus among the authors before inclusion in our analysis. We excluded papers that did not handle ESFC or focused on production methods or technical aspects of ESFC. In line with our focus on high-income countries, we also excluded papers that solely focused on emerging markets (e.g., Brazil, Thailand). We ended up with 339 papers illustrating the literature on ESFC. This set of papers was then read to give us a fair indication whether and how the components in our framework have been tackled in research and which gaps need to be closed. To strengthen the discussion, we complemented these selected papers with papers that offer general theoretical insights from outside the ESFC domain but can be applied to it. The current paper thus offers a structured, narrative review of literature relevant to ESFC.

### Step 1: Positive Value

#### Conceptual Background

The end state at which a behavior is directed needs to have a positive value. If an end state is not valued, it will not be pursued. Consumers will engage in ESFC only if they value the environment and/or the improvement of its state. Hence, an important first step into encouraging ESFC is to promote environmental values. Environmental values encompass the goal to act in an environmental friendly manner, for instance by purchasing environmental sustainable (food) products (Bardi and Schwartz, 2003). The relation between valuing the environment and environmental sustainable consumption has been established in several studies for non-food (e.g., Haws et al., 2014) and food products (e.g., Vermeir and Verbeke, 2008). For example, de Boer et al. (2013) observed a negative relationship between Dutch people’s endorsement of care for nature as a value and current meat consumption as well as the willingness to eat one or more meals without meat every week in the future.

Data suggest that the proportion of consumers who engage in environmentally sustainable consumption for environmental reasons in particular, is relatively limited. For example, Mullee et al. (2017) examined the reasons to reduce future meat consumption in Belgium and found that as little as 11.1% of the omnivores and flexitarians would consider eating a more vegetarian diet because of the impact of meat on the environment/climate. Yet, individuals can engage in ESFC for other reasons than its positive effect on the environment. For example, people can buy environmentally sustainable products for functional, social, ethical or emotional reasons (Mullee et al., 2017; Sangroya and Nayak, 2017) like price and health (e.g., nutritional value, food safety) perceptions, sensory appeal (e.g., taste), animal welfare and supporting the local economy (Hughner et al., 2007; Bauer et al., 2013; Banovic et al., 2019).

#### Interventions to Activate Positive Value for Individuals Who Value the Environment

Even if individuals value the environment, it may still be beneficial to increase the salience of these environmental values at the point of decision making to ensure they positively affect decision making (e.g., Verplanken and Holland, 2002; Dijksterhuis et al., 2005; Verplanken et al., 2008). Several ways have been identified to activate environmentally sustainable values, including priming (Verplanken et al., 2009; Hahnel et al., 2014) and the activation of personal norms (de Groot and Steg, 2009).

##### Prime environmental values

For people who value environmental goals, priming environmental values (i.e., increasing their accessibility or the ease with which they can be retrieved from memory), or activating other associated constructs in memory (Wheeler et al., 2005), can be used to make environmental values more salient (Chartrand and Bargh, 1996). Loebnitz and Aschemann-Witzel (2016) primed environmental values by instructing their participants to think about five environmental values (e.g., preserving nature, caring for future generations). Once a motivation to pursue a value is activated, goal-directed cognitive and behavioral processes may follow spontaneously and result in goal-congruent choices (e.g., Bargh, 1990), especially when these values are personally relevant (Fazio, 2001). Priming environmental values could also help people to forgo immediate rewards in the present for longer-term payoffs in the future (self-regulation, Baumeister et al., 1998). In a food context, priming environmental values increases the importance of environmental friendly product attributes (Loebnitz et al., 2015) and increases product (health and quality) expectations for organic-labeled food items (Loebnitz and Aschemann-Witzel, 2016).

##### Activate personal norms

In addition to priming, activating personal norms (i.e., self-expectations that are based on internalized values, Schwartz, 1977) can indirectly activate environmental values (cf. the Value–Belief–Norm theory; Stern, 2000) and consequently trigger pro-environmental behavior (Lindenberg and Steg, 2007; Steg et al., 2014) for individuals for whom environmental values are central to the self. To activate personal norms one’s awareness of environmental issues can be increased, for example, by pointing out the environmental impact of behavior and the fact that these consequences can be averted. As a surplus, providing environmental knowledge to people who highly value the environment strengthens these values. Zepeda and Deal (2009) suggest that increased knowledge on organic and local food production reinforces existing values, which -via changed attitudes- support environmentally sustainable purchase behavior (i.e., local food). Personal norms can also be activated by increasing feelings of responsibility (de Groot and Steg, 2009), by asking people to think about behaviors associated with strong personal norms or by making people solve a word puzzle including sentences like “give your best work,” or “meet your own target” which primes personal norms (Chandon et al., 2011). We propose the following Future Research (FR) Question:

→ FR 1. To what extent does activating personal norms strengthen environmental values?

#### Interventions Targeting People Who Do Not Value the Environment

The interventions discussed in the previous section target people who already have (latent) pro-environmental values. For people who do not have pro-environmental values, other interventions are required. However, most interventions mentioned in this section may also have a positive impact on people who have positive pro-environmental values. If consumers do not value the environment, we distinguish between four possible courses of action: (1) strengthening the relative value of the environment through persuasion; (2) promoting ESFC through harnessing goals unrelated to ESFC; (3) strengthening the relative value of ESFC through punishment of undesirable outcomes of non-ESFC; and (4) evoking immediate behavior.

##### Strengthening the relative value of the environment through persuasion

Values do not change overnight. As people more clearly experience local impacts and recognize environmental change, the segment of society that sees climate change as a threat is expected to gradually grow (Marshall et al., 2019). To speed up this process, the value of the environment could be strengthened by communication messages focusing on (1) mental imagery of the (negative) consequences of (not) acting sustainably, (2) positive cueing, (3) social norms, or (4) issue severity.

###### Evoke mental imagery

Gregory and Leo (2003) provide evidence that personal involvement develops when individuals become aware of the consequences of their behavior. Making people think about the future benefits of the sustainable action could make it more desirable in the present (Reczek et al., 2018). When the aversive consequences of failing a subgoal (e.g., failing to recycle a newspaper) are shown, the perceived importance of the related end goal (e.g., sustaining the natural environment) increases (Devezer et al., 2014). Communications focusing on the negative consequences of failing an environmental subgoal can make the benefits of pro-environmental behaviors more concrete, visible and feasible so that they outweigh the costs of sustainable behavior (Guthrie et al., 2015). Devezer et al. (2014) suggest that individuals could be stimulated to find environmental values more important when they can easily visualize this end goal. Messages should explain precisely how a behavior change should occur (White et al., 2011) and what the outcome could be, and this explanation should be vivid and involving without having vivid and distracting additional information (Bator and Cialdini, 2000). Also, messages that relate immediate impact of pro-environmental behavior to a specific location (Scannell and Gifford, 2013) or to the self (Spence et al., 2012; Reczek et al., 2018) can make environmental sustainable actions more tangible and relevant. Messages could encourage individuals explicitly to mentally simulate the portrayed outcome (e.g., “imagine a world without pollution”), present outcomes in a concrete way (e.g., by showing a clear sky free of smog), use easily interpretable verbal stimuli (e.g., “help build a clean world with clean skies”), or stimulate the immediate interpretation and elaboration of the presented outcome (e.g., “Think right now on the consequences of …”; also see “mental contrasting,” discussed under “Competing Goals”). What is important here, is concretization of abstract risks, since this motivates action more than analytic understanding (Marx et al., 2007). An important related question pertains to the extent to which environmental issues can be represented by concrete, countable, tangible representations (e.g., a pile of waste, a cloud of exhaust, a deforested area,…).

→ FR 2. Which verbalizations and visualizing techniques increase the value of the environment?

###### Use positive cueing

Cornelissen et al. (2008) use positive cueing to engender pro-environmental self-perceptions and increase the feeling of moral obligation to act pro-environmentally. People often dismiss more common ecological behaviors like avoiding food waste or buying seasonal produce as non-diagnostic for their environmental conscious self-image and hence they fail to see themselves as environmentally conscious consumers (Cornelissen et al., 2008). Positive cueing entails cueing common environmental behaviors like avoid wasting food and buying seasonal produce as environmental so that this behavior becomes diagnostic for one’s environmental conscious self-image (Cornelissen et al., 2008). This can be done, for instance, by framing common behaviors as pro-environmental in a questionnaire (e.g., by asking questions like “Which of the following pro-environmental actions do you usually engage in?”). This leads consumers to view themselves as concerned with the environment, subsequently resulting in more environmentally friendly food choices. Positive cueing could boost the importance of environmental values as it makes people see themselves as “someone who is willing to do an effort for the environment” and hence internally motivated to act upon that self-perception (Osbaldiston and Sheldon, 2003).

→ FR. 3. When does positive cueing increase the value of the environment?

###### Activate social norms

Social norms about eating have a powerful effect on both food choice and amounts consumed (Higgs, 2015). Social norms (i.e., the rules that guide, regulate and proscribe social behavior in particular contexts, Burchell et al., 2013) show people how they “should” behave. Behavioral choices are based on evaluations about what is right or wrong (Lindenberg and Steg, 2007). By showing social norm messages, social norms can be activated (Schwartz, 1977) and feelings of moral obligation could set in. These feelings are related to the beliefs and values that people adhere to (cf. Value-Belief-Norm theory, Stern, 2000) which suggests that environmental values could increase when seeing messages that activate social norms. Note, however, that social norms can also lead to ESFC when ESFC is seen as a way to achieve the alternative goal of behaving in line with social norms (Moors et al., 2017). Interventions using cues that suggest specific social norms or provide feedback on one’s own behavior in comparison to the behavior of relevant others have been shown to effectively influence pro-environmental consumption behavior (Biel and Thøgersen, 2007; White et al., 2009; Kormos and Gifford, 2014; Onel, 2017) especially in social or public situations (Griskevicius et al., 2010; White et al., 2014). Demarque et al. (2015), for example, found that shoppers in an experimental online store bought more eco-labeled products when they received information on how many percent of previous shoppers bought ecological products (cf. norm activation model, Cialdini, 2003). As a downside, using a descriptive norm message could cause a boomerang effect if people think that non-environmental friendly behavior is the norm (Cornelissen et al., 2008). Hence, interventions that activate social norms could both encourage people to value or disvalue the environment.

→ FR 4. To what extent and when do interventions activating social norms affect the value of the environment?

###### Increase issue severity

Obermiller (1995) found that presenting a problem as severe or threatening should increase attention to messages and result in favorable attitudes toward the actions proposed in that message, especially when an environmental issue is considered as relatively unimportant. He suggests that (environmental) concerns can increase for people who value the environment less and who believe the claims put forward in the threat message. Relatedly, Cucchiara et al. (2015) found that interventions increasing awareness that the environment is under threat especially impact consumers who believe that their consumption choices will not make a difference and who minimize the negative environmental impact of human consumption practices. Furthermore, optimal results were found when both the severity of the problem was highlighted and information how to act upon it (cf. threat and coping appraisal, protection motivation theory, Rogers, 1975). On the other hand, research on fear appeals shows that they may be ineffective (Hastings et al., 1995), as evoking too much fear can have opposite effects. O’Neill and Nicholson-Cole (2009) also suggest that personal engagement with an environmental issue can decrease when confronted with fearful representations of climate change. Future research could investigate which degree of fear can increase the value of the environment and whether information on how to solve the issue in the threat appeal should be available to optimally enhance value of the environment. If not, inducing fear could possibly evoke defense reactions that negatively affect the value of the environment and environmentally sustainable food choices.

→ FR 5. To what extent and under what conditions can fear appeals enhance the value of the environment?

Different interventions can strengthen the relative value of the environment. Yet, several mechanisms may interact either negatively (e.g., fear appeals and positive cueing) or positively (e.g., positive cueing and descriptive social norms, i.e., norms describing what people usually do, or injunctive social norms, i.e., norms that indicate what people ought to do).

→ FR 6. What is the interplay between interventions strengthening the relative value of the environment and how do they call upon people with either negative/absent/latent/salient pro-environmental values.

##### Promoting ESFC through harnessing other desirable outcomes

If people do not value the environment (much), or if they have other, more dominant values, they can also be triggered into buying sustainable products as a way of attaining goals that they value more positively (e.g., buying more expensive organic food as a status symbol; van der Wal et al., 2016). Hence, the goal-directed perspective captures the fact that similar types of sustainable consumption can be motivated by different goals (e.g., saving money, achieving higher social status, eating healthier, acting ethically, …). As such, tapping into personal rather than environmental benefits could induce greener purchasing behavior in some instances (White and Peloza, 2009; Gifford, 2011; Green and Peloza, 2014; Feldmann and Hamm, 2015) as it may demonstrate that ESFC is consistent with values, goals and beliefs that people who do not value the environment (much) adhere to Lindenberg and Steg (2007), Von Borgstede et al. (2014).

###### Highlight sensory benefits

People may seek sensory benefits from ESFC. Superior sensory appeal and taste are influential drivers for buying organic products for example (Renko et al., 2011). Research also found that sensation seeking is an important antecedent for acceptance of novel products (e.g., Lammers et al., 2019). The dominant approach to market novel food products is highlighting health or environmental benefits (Berger et al., 2018). This is surprising because emphasizing hedonic aspects like, for instance, the taste of insect-based foods would fit better with the underlying sensation seeking motive, and would also be more effective (Berger et al., 2018). Hedonic claims also outperformed health claims for atypically shaped vegetables of which taste expectations and naturalness perceptions are often negatively evaluated (Turnwald et al., 2017). Hedonic claims could also decrease feelings of disgust and consideration of unnaturalness that are an important barrier to consume cultured meat (Verbeke et al., 2015a; Anderson et al., 2019; Circus and Robison, 2019; Shaw and Iomaire, 2019). However, overruling spontaneous negative feelings (disgust, fear, …) triggered by (visually) unappealing foods will be difficult. This calls for more research into the potential of interventions to stress the pleasurable sensory aspects of environmental friendly foods such as misshapen vegetables, cultured meat or insect-based foods. Current insights highlight, for example, the potential of anthropomorphic techniques (e.g., displaying misshapen produce with a smiling face and presenting shape abnormalities as body parts) to activate pleasurable feelings and stimulate the consumption of visually unappealing food (Cooremans and Geuens, 2019).

→ FR 7. To what extent, when and why does stressing sensory aspects or using anthropomorphic techniques increase ESFC?

###### Emphasize health benefits

Since people could engage in green consumption as a way to improve health (Bostrom et al., 2013; Howell, 2013; Bullock et al., 2017; Witek, 2017), future research can test interventions highlighting health benefits of ESFC. Framing ESFC as a health issue could even induce feelings of hope (Myers et al., 2012) which can increase ESFC (Feldman and Hart, 2018). Health-related concerns are particularly relevant drivers of organic food consumption (Janssen, 2018) and reduced meat consumption (Malek et al., 2019). For now, there is little evidence on consumer perceptions of health-related beliefs concerning insect-based foods and seasonal produce.

→ FR 8. Under which conditions can interventions stressing health benefits increase ESFC?

###### Point out value for money

Although price concerns can be an important barrier to ESFC (Verain et al., 2012; Aschemann-Witzel and Zielke, 2017), the perceived value of these products can also increase as higher prices can indicate higher “acceptable quality” (Sangroya and Nayak, 2017). Higher prices can also signal trustworthiness (Gottschalk and Leistner, 2013). Hence interventions could stress the utilities and benefits individuals can obtain from environmentally sustainable products despite possible price premiums.

→ FR 9. To what extent can ESFC be increased by stressing positive signals related to higher prices?

###### Point out other non-environmental benefits

In addition to sensory, health or value-related benefits, other non-environmental benefits might be linked to ESFC. For example, if hedonic goals are prevalent, messages could demonstrate how acting pro-environmentally can make people feel good. Also, Tezer and Bodur (2020) show that people who use a green product without being responsible or accountable for the decision to use the product (for example, they get recycled 3D glasses in the cinema) experience higher enjoyment of the accompanying consumption experience. This “green consumption effect” is driven by an increase in perceived social worth which results in a warm glow. Future research needs to investigate in a structured way which different benefits can act as a “feel good” factor that adds value to the overall product (Wong et al., 1996) and can hence be potential reasons for engaging in ESFC. Also the role of social norms could be examined from this perspective as social norms relate to the goal of getting approval from others.

→ FR 10. Which non pro-environmental (perceived) benefits can act as potential reasons for engaging in ESFC?

###### Provide incentives

Financial incentives can lift the price barrier that is often limiting ESFC. Both financial and non-financial incentives have been shown to be effective in changing eating patterns (Purnell et al., 2014; De Marchi et al., 2019). Caird et al. (2008), Lin and Huang (2012) found that discounts, incentives and subsidies can enable individuals to participate in environmentally friendly consumption. Non-financial incentives (e.g., gadgets) can also be successful in increasing vegetable consumption in a sample of 11−14 year-old children, an effect that can even persist several weeks after the provision of the incentives ends (De Marchi et al., 2019). In addition, van Horen et al. (2018) showed in a student sample that competition can be an incentive to motivate pro-selves (i.e., people who are more concerned about taking care of the self and hence less engaged with climate, Corner and Randall, 2011) and pro-socials (i.e., people who are socially conscious and already committed to the sustainability agenda, Balliet et al., 2009) to act in a pro-environmental way by having them compete to realize pro-environmental objectives. The success of this approach is explained by the fact that pro-socials are motivated to act in a pro-environmental way (regardless of the competition), while pro-selves are motivated by competition (regardless of the pro-environmental aspect). Other research argues that the mere use of economic incentives (i.e., material rewards) is unable to lead to a sustained diffusion of eco-friendly alternatives in the market, because purchasing behavior returns to baseline levels after the reinforcement is terminated (Cairns et al., 2010; Oliver and Rosen, 2010; Steg et al., 2014). Also intrinsic motivation to engage in a behavior can be reduced when this behavior is incentivized (Gneezy et al., 2011; Kamenika, 2012), by which incentives may decrease food preferences (Newman and Taylor, 1992). This leads to an important paradox that requires further investigation, as on the one hand, incentives may decrease pro-environmental food preferences, but on the other hand they can lower the price barrier often limiting ESFC.

→ FR 11. To what extent can ESFC be increased by providing (non-) financial incentives?

##### Strengthening the relative value of ESFC through punishment of undesirable outcomes of non-ESFC

###### Impose taxes

Even when people do not value the environment, they could be triggered into buying sustainable products, for instance through the imposition of taxes on non-ecological alternatives. Hagmann et al. (2019) recently suggest that taxes and subsidies could be the most effective policies for reducing carbon emissions. Research indicates that meat carbon consumption taxes have the potential to reduce household demand for meat products, with greenhouse gas emission reduction estimates in the range of 10.5% (in Scotland; Chalmers et al., 2016) to 12% (for a tax on meat and dairy in Sweden; Säll and Gren, 2015). Nordhaus (2001) also suggested that policymakers should consider harmonized environmental taxes on carbon as powerful tools for coordinating policies and slowing climate change. Taxes can especially be effective in domains that involve strong habits (Krause, 2009) but they can induce negative effect and defense responses (e.g., Steg and Vlek, 2009).

Interestingly, Hagmann et al. (2019) show that support for a carbon tax diminishes when individuals also get the possibility to choose for a green nudge (see “Interventions to Evoke Immediate Behavior” for an explanation of a nudge) even if people know that this nudge is less effective than a tax (Hagmann et al., 2019). However, informing the public that nudges are not a substitute for more substantive policies, even if they are cost-effective, increases support for taxes without diminishing support for nudging interventions (Hagmann et al., 2019).

→ FR 12. Which conditions or interventions can create solid consumer support for taxes on non-ecological alternatives?

#### Interventions to Evoke Immediate Behavior

##### Nudge

Instead of explicitly increasing the (salience of the) value of the environment or promoting ESFC through harnessing other desirable outcomes of environmentally sustainable food products, people can also be nudged into choosing an environmentally sustainable food product in the context in which they make their decision, irrespective of their values. Nudging aims to change people’s behavior in a predictable way without forbidding any options or significantly changing their economic incentives (Thaler and Sunstein, 2008). Nudging does not necessarily aim to change the importance of individual’s values or behavior, but can also evoke immediate behavior without increasing the value of sustainable consumption. Since food choices are often guided by fast, automatic and/or cognitively effortless responses to environmental stimuli, nudging interventions that urge action without necessarily evoking thoughts about value-action discrepancies could be an easy and cheap solution. Changing the decision context (i.e., by optimizing the choice architecture, Thaler, 2018), can change the salient cues that affect cognitive responses to a situation and the resulting behavior. By adapting elements in the choice environment such as the way products are positioned, their visibility or packaging, choices are affected.

Several studies have shown the effectiveness of nudging interventions to steer individuals to more ESFC (Ferrari et al., 2019), for example, by decreasing portion sizes of less sustainable meat (Vandenbroele et al., 2018), or by increasing visibility of meat substitutes (Vandenbroele et al., 2020a) or more sustainable meat (Coucke et al., 2019). Nudges for ESFC at the point of purchase can be categorized according to whether the nudge exerts an influence on consumers’ cognition (i.e., consumer knowledge), affect (i.e., consumers’ feelings) or behavior (i.e., motor responses) (Cadario and Chandon, 2019), as reviewed in Vandenbroele et al. (2020b). Vandenbroele et al. (2020b) discuss several future research areas that could be worthwhile investigating like the effect of interventions increasing the availability perceptions of environmentally sustainable food products. Increasing the perceived availability of eco-labeled products might not only trigger immediate choice but could also influence goal-pursuit, for instance by increasing public awareness of the environmental impact associated with food production or even increasing environmental values. More generally, it is important to realize that nudging interventions could influence behavior via their impact on goal-pursuit.

→ FR 13. Which nudging interventions positively affect ESFC; to what extent do nudging interventions influence ESFC by increasing public awareness or environmental values?

### Step 2: Perceived Discrepancy

#### Conceptual Background

People will engage in goal-directed behavior only when they perceive a discrepancy between the current state and the end state that they value (Moors et al., 2017). For instance, even people who value the environment are not likely to engage in ESFC if they believe that the environment (or environmental aspects they consider to be important) is not under threat. For instance, people might dismiss global warming as a threat because they believe that it will improve the climate at the location where they live.

Gifford (2011) found that denial of climate change can be led by fear. Terror management theory (e.g., Goldenberg et al., 2000) suggests that people may deny this problem because it is a reminder of their mortality (Vess and Arndt, 2008). de Boer et al. (2013) showed the relation between the experience of a discrepancy (i.e., an environment that is under threat) and sustainable food consumption with regard to meat consumption (de Boer et al., 2013). The more consumers showed climate skepticism the less they were willing to reduce their meat consumption.

#### Interventions That Can Increase Perceived Discrepancy

##### Increase self-monitoring

Previous research has shown that monitoring progress toward a goal has a robust effect on goal attainment as it identifies the discrepancy between the current state and the desired state (Harkin et al., 2016). It enables people to identify how best to allocate effort among salient goals (Carver and Scheier, 1981; Louro et al., 2007) and whether they should exert more effort or self-control (Myrseth and Fishbach, 2009). Self-monitoring could also make people less capable of avoiding information which indicates that they were not progressing toward their goal (Webb et al., 2013). A way to increase self-monitoring is to ask a person to keep a diary of their environmental sustainable consumption or compare their current ESFC to their previous ESFC (Harkin et al., 2016). We note that self-monitoring has received more attention as an intervention in a health context (Burke et al., 2011) than in the context of ESFC, probably because environmental food related outcomes are harder to operationalize.

##### Decrease skepticism

Further research could identify ways to decrease skepticism (disbelief) toward environmental issues by, for example, enhancing perceptions of collective efficacy (Fritsche et al., 2018). Also, since skepticism has been linked to specific social groups (e.g., political conservatives), interventions could be aimed at framing environmental goals as compatible with the goals of these groups (e.g., focus on environmental action as an act of conservation) or by motivating people to identify with a self-identity at a more collective level like “humanity” (thus superseding identification with the skeptical group) (Fritsche et al., 2018). An important question in this regard is whether social groups that tend to be skeptical are open to such collective-level identifications as these types of identification may be more in line with progressive, prosocial self-perceptions, and may consequently backfire.

→ FR 14. Which interventions decrease skepticism toward environmental issues?

##### Decrease justifications

In general, people prefer making choices that can be easily justified (e.g., Shafir et al., 1993). People sometimes use system justification (i.e., the tendency to defend and justify the societal status quo) which results in ignoring or denying environmental problems and perpetuating harmful behaviors (Feygina et al., 2010). System justification can be reduced by portraying the necessary increase in ESFC as being part of the system rather than a consequence of the system (Feygina et al., 2010). Other justification mechanisms include perceived inequity (“why should I change if others won’t change?” Gifford, 2011), uncertainty (disregarding likelihood of climate change by phrasing “it is likely” rather than “it will happen”; Budescu et al., 2009); judgmental discounting (“it is worse in places other than my own”; Gifford et al., 2009); optimism bias (“my environment will not deteriorate as much as another place”; Gifford et al., 2009); believe in supra-human powers (“Mother nature or God will save us”; Mortreux and Barnett, 2009); technosalvation (“new technologies will save us”; Lorenzoni et al., 2007) and denial (“human activity does not cause climate change”; McCright and Dunlap, 2010). In the context of meat consumption, there is solid evidence showing that meat-eaters engage in a variety of psychological defense mechanisms to justify their behavior (e.g., Rothgerber, 2013). Some meat-eaters argue, for example, that “meat is essential for strong muscles” (i.e., health justification) or that “God intended for us to eat meat” (i.e., religious justification). Each of these justifications may help to minimize the importance of reducing meat intake, even in individuals who otherwise attach great value to the environment, the climate, healthy eating, and/or animal welfare. Hence, the use of these justifications should be minimized.

→ FR15. To what extent and how can justifications be minimized?

### Step 3: Goal Intention

#### Conceptual Background

When confronted with a perceived discrepancy between the desired versus the actual state of the environment, several responses are possible. Ideally, consumers may decide that they need to act to reduce the discrepancy. People could form an intention to act on their experienced discrepancy or a “goal intention” thereby committing themselves to the execution of actions needed to achieve this goal (Bagozzi and Dholakia, 1999). A goal commitment entails the self-realization that actions are required to achieve the goal but does not specify the actions that need to be executed for goal achievement (Bagozzi and Dholakia, 1999). A multitude of research focused on the factors influencing goal intentions (e.g., Perugini and Conner, 2000; Armitage and Conner, 2001; Rodgers et al., 2010). Bagozzi and Kimmel (1995) compare several of these theories on their ability to predict intentions and behavior. Several researchers furthermore investigate what factors influence intentions and behavior in a sustainable (food) context (e.g., Hines et al., 1987; Axelrod and Lehman, 1993; Mainieri et al., 1997; Tanner, 1999; Han and Hansen, 2012; Tripathi and Singh, 2016). But alternatively to intending to act on their goal, consumers may question the perceived discrepancy (e.g., “Is this threat really that big?”), by disengaging from the issue, for example by devaluing the need for a healthy environment at this moment in time (e.g., “I don’t care because I’ll be dead by the time the problems really start”; “It is OK to continue polluting because future generations will manage to create technology to clean up”). They might also change their beliefs about the necessity of acting by, for example, believing people who claim that the problems with the environment are not that bad anyway. People often exhibit self-defensive reactions when they learn that their behavior can have negative environmental impact (Feygina et al., 2010) and display motivated biases like the tendency to seek out information that confirms preexisting views (Weber, 2016). Experiencing a discrepancy between one’s actual and desired state may cause cognitive dissonance (i.e., experiencing discomfort when behaving inconsistently with one’s attitudes, Festinger, 1957). Cognitive dissonance is often more easily resolved by changing one’s mind (“eating red meat is not really causing the problem”) than by changing one’s behavior (by eating less or no meat). For those who do change their beliefs and hence no longer experience dissonance, it is imperative that this discrepancy is re-evoked. This component of the framework captures why it is important to educate people about the ways in which the environment is under threat and why those threats matter.

#### Interventions to Make People Intend to Act on Their Goal Intention

In case the value of the environment is questioned, interventions mentioned in “Interventions Targeting People Who Do Not Value the Environment” apply, whereas in case consumers question the discrepancy between the actual and desired state of the environment, we refer to the suggestions discussed in “Interventions That Can Increase Perceived Discrepancy.”

##### Increase cognitive dissonance

When people react to perceived discrepancy by changing the belief that it is necessary to act, interventions could be aimed at increasing cognitive dissonance and, hence, the likelihood that consumers access pre-existing beliefs or attitudes that promote sustainable food consumption (Osbaldiston and Schott, 2012). Cognitive dissonance can be increased by questioning one’s moral standards (cf. increasing salience of the discrepancy between one’s norms and one’s behavior, Aronson and Carlsmith, 1962; Thøgersen, 2004) by for example letting people make a speech for engaging in pro-environmental behavior and then remind people of the times they failed to engage in pro-environmental behavior (cf. Aronson et al., 1991), or pointing out to people that they use biased assimilation (i.e., denying the validity of information that is inconsistent with an existing belief; Ahluwalia, 2000).

→ FR 16. To what extent can increasing cognitive dissonance increase goal intention?

##### Increase personal responsibility

Very little research on sustainable food choices has addressed the issue of personal responsibility. While consumers may be aware and convinced of the necessity to adopt environmentally friendly behavior, in order for them to act they may still need to be convinced of their personal role in solving environmental problems. Only few studies on sustainable food choice have explored potential interventions in this respect. As an exception, Antonetti and Maklan (2014) focus on the self-conscious emotions “guilt” and “pride” and find that experiencing guilt or pride makes consumers see themselves as contributing to solving environmental issues. These feelings reduce the use of neutralization techniques that would otherwise rationalize away consumers’ responsibility. Hence interventions could stress, for example, guilt or pride to evoke environmental sustainable choices. Related to this, individuals can resolve their internal discrepancy using coping mechanisms (i.e., “cognitive and behavioral efforts made to manage external and internal demands and conflicts among them”) (Lazarus and Folkman, 1984). Moruzzi and Sirieix (2015) identify coping mechanisms in the context of sustainable consumption where French and Italian consumers either ignore, neglect or distance themselves from sustainable products or labels or search for labels or information from trusted known sources (such as relying on word-of-mouth spread by acquaintances). Hence, interventions could point out to consumers that they use coping mechanisms in order to act on their discrepancy.

→ FR 17. To what extent can evoking guilt or pride or stressing coping mechanisms increase an individual’s sense of personal responsibility and goal intention?

##### Counteract compensatory behavior

Consumers may also show reduced goal intention once they have already engaged in sustainable behavior. That is, consumers have a tendency to compensate sustainable behavior in one domain with increased unsustainable behavior in the same or another domain (Otto et al., 2014), in part because performing a sustainable act can make people feel less obliged to perform subsequent sustainable choices (Thøgersen and Olander, 2003) and can license unsustainable behavior (Nilsson et al., 2017). Consumers have been found to endorse compensatory green beliefs (Kaklamanou et al., 2015; Hope et al., 2017), such as “You do not need to worry about which country your food comes from if you use energy-efficient appliances in the home” or “Composting food waste can make up for buying imported food.”

A related (yet distinct) phenomenon is the negative footprint illusion: even though adding an ecological to a non-ecological food product increases the total footprint of the menu, consumers sometimes mistakenly estimate the total footprint of the combination of the ecological and the non-ecological product lower than the same non-ecological product alone (Gorissen and Weijters, 2016). So, for instance, consumers may erroneously have the impression that adding an organic apple to a beef burger menu reduces the footprint of their overall menu.

An important topic for future research relates also to the question how compensatory beliefs, licensing, and the negative footprint illusion can be countered. After all, if consumers engage in ESFC only to compensate that behavior afterward by indulging in more unsustainable behavior in some other decision, little has been gained. It is currently not sufficiently clear how these phenomena can be successfully countered and more research is needed to establish under what conditions they occur.

→ FR 18. How can compensatory beliefs, licensing and the negative footprint illusion be countered?

In addition, more indirect defense mechanisms may be at play. For example, (female) meat-eaters tend to underestimate their objective meat intake as a way to minimize one’s own impact on climate change, and hence underestimate the need for personal behavioral change (Rothgerber, 2019). It is well-documented that people tend to interpret evidence in a self-serving manner, which leads people to exaggerate their contribution to environmental protection (Pieters et al., 1998) but minimize their contribution to environmental problems. These direct and indirect defense mechanisms devalue the outcome (cf. “Interventions Targeting People Who Do Not Value the Environment”), question the discrepancy between the actual and desired state of the environment (cf. “Step 2: Perceived Discrepancy”), or reduce the goal intention itself.

→ FR 19. Which indirect defense mechanisms do people use and how can they be reduced?

### Step 4: Behavioral Intention

#### Conceptual Background

When people decide to act on their goal intention in order to reduce a perceived discrepancy between an actual state and a desired end state, they still need to decide *how* to act. When people have selected a behavior that they intend to perform, they are said to have formed a behavioral intention. It is typically assumed that people will choose an action of which they expect that it will bring them closer to the desired end state (Kruglanski et al., 2015; Moors et al., 2017). In those cases where ESFC is driven by pro-environmental goals, sustainable consumer behavior therefore crucially depends on subjective beliefs about which behaviors promote or burden the environment. Hence, in order to encourage consumer behavior that is objectively sustainable, it is vital to promote correct expectancies about the environmental impact (but also other effects) of specific consumer choices. This can primarily be done (a) by promoting general background knowledge and/or (b) by providing specific informational cues at the point of purchase.

#### Interventions to Guide and Strengthen Behavioral Intentions

##### Increase background knowledge

In terms of general background knowledge, it is key to align expectancies related to environmental effects of food choices as well as potential side-effects of ESFC with reality. As to the latter (expected side-effects of ESFC), perceived risk has been identified as a deterrent to the adoption of eco-consumption (Boivin et al., 2011). For example, some individuals associate eating vegan with a physical risk (e.g., “I will not get all necessary nutrients”) or social risk (e.g., “others will talk about me”). Informing people about the minimal risks involved in ESFC can reassure them and trigger sustainable behavior.

Some research has identified inaccurate or incomplete environmental expectancies. For instance, many consumers are unaware of the impact of eating meat on the environment (e.g., only about a third of respondents linked cattle farming to climate change; Hartmann and Siegrist, 2017; Mullee et al., 2017). Even if these consumers care for the environment, they will not reduce their meat consumption. Relatedly, lack of environment-related information is a key hurdle in the purchase of insect-based food products (Lammers et al., 2019). Hence, for individuals who do not have correct beliefs about the environmental impact of certain food choices, interventions should be set up to increase their knowledge about which behavior is sustainable (Gifford and Nilsson, 2014).

Importantly, past research needs to be interpreted with caution, as consumer awareness may be rapidly evolving. For instance, in a large-scale longitudinal panel study, Siegrist et al. (2015) found that participants evaluated eating less meat (maximum of once or twice per week) as substantially more beneficial for the environment in 2014 compared with 2010, and it is plausible that consumer perceptions have continued to shift since then.

##### Provide specific informational cues

One important type of informational intervention provides cues on the environmental impact of food products at the point of purchase (or on product packaging). This type of intervention includes the use of green claims and eco-labels. As to the former, consumers prefer products with green claims over those with neutral (control) claims, and products with emotional green claims over those with rational green claims, even though this effect is moderated by participants’ environmental commitment, information processing ability and by distraction (Aagerup et al., 2019).

As a somewhat more structured type of intervention, various eco-labels have emerged with the aim of communicating the ecological merits of products (Delmas and Lessem, 2017; Yokessa and Marette, 2019). Eco-labels using logos have been found to capture visual attention more than text (Rihn et al., 2019). Familiar and trusted labels generate positive perceptions (Cornelissen et al., 2008; Sirieix et al., 2013), and adding eco-labels to novel, sustainable food products has been found to increase choice likelihood (in the context of aquaculture foods; Schacht et al., 2010; Banovic et al., 2019). But even though consumers’ understanding of a set of selected labels (Fair Trade, Rainforest Alliance, Carbon Footprint, and Animal Welfare) is good, these labels do not play a major role in consumers’ food choices (Grunert et al., 2014). More worryingly, consumers face an ever increasing number of sustainable food labels, some of which may be complementary, while others add to the growing competition of product information in consumers’ minds (Sirieix et al., 2013), resulting in consumer confusion, distrust, and dissatisfaction (Moon et al., 2017). The complexity and the proliferation of eco-labels thus hamper their efficiency in promoting ESFC (Yokessa and Marette, 2019).

Research on the effectiveness of eco-labeling points toward the following recommendations. First, consumers in general (i.e., in a context not limited to food) attach credibility to ecolabels that they trust, which typically includes ecolabels certified by third parties like governments or environmental NGOs (Darnall et al., 2018). Consistent with this, eco-labeling in the context of organic coffee is more impactful when certified by a public authority (Thøgersen and Nielsen, 2016). Second, labeling choice options that should be avoided (i.e., using a negative frame) is likely more effective than only labeling the environmentally preferable options (Grankvist et al., 2004; Van Dam and De Jonge, 2015). Third, eco-labels work best if they are informative yet easy to interpret. Traffic light labels (with green-yellow-red codes indicating good to bad environmental friendliness) have been found to be effective in grocery shopping in general (Wiese et al., 2015), as well as in specific categories like coffee (Thøgersen and Nielsen, 2016) and seafood (Hallstein and Villas-Boas, 2013).

A lot of research has studied consumer responses to different eco-labels on food products, but several important research questions have not been addressed in sufficient detail. For one, the organic label in particular has received a lot of research attention (Bauer et al., 2013; van Doorn and Verhoef, 2015; Aschemann-Witzel and Zielke, 2017). However, organic labeling has particular effects, like halo effects suggesting a host of personal benefits to the consumer (health, taste, safety, nutritional value, etc.). Such halo effects are unlikely to be generalizable to other eco-information schemes that are often more closely aligned with primary environmental outcomes, like carbon labeling (Röös and Tjärnemo, 2011). Eco-labeling research needs to investigate which insights gleaned from organic labeling research can be extrapolated to other labels. For this quest to be successful, it will be necessary to define a typology of eco-labels that allows researchers to systematically link eco-label characteristics to eco-label effects.

→ FR 20. Which typology of labels can bring structure to the labeling literature?

Relatedly, practitioners and researchers have also studied alternative eco-information schemes that employ ratings or metrics, including carbon footprint labeling (Lee et al., 2012) and food miles (MacGregor and Vorley, 2006; Schnell, 2013). However, given the dearth of comparative research, it is not clear which types of eco-information schemes are more effective.

→ FR 21. What is the moderating effect of labeling characteristics on their effectiveness?

Some product attributes that are environmentally relevant have not been consistently communicated to consumers and (partly as a result) have not been researched in a very systematic way. For instance, there is currently a lack of a standardized labeling approach for identifying local food, which makes it difficult for consumers to identify local food products (Feldmann and Hamm, 2015).

→ FR 22. How to effectively communicate (multiple) environmentally relevant product attributes (other than organic)?

Consumers have been found to be confused by the presence of multiple labels (Moon et al., 2017). This raises the question how different types of (eco-related) labels interact. In one interesting initial study in this direction, Sörqvist et al. (2016) explored how consumers in a Swedish and a United Kingdom sample respond to combinations of eco-labeling and Genetically Modified Organism (GMO) labeling in terms of judgments of taste, health consequences and willingness to pay for raisins, and found that the GMO-label removes the psychological benefits of the eco-label (especially among Swedish participants). Thøgersen et al. (2017) reviewed the literature to shed light on the possible interaction between the effects of organic and country-of-origin labeling on consumers’ food preferences and choices. Building on this type of research, more studies are needed on the joint use of different types of eco-labels with other types of (eco-) labels. Relatedly, consumers perceive better product quality and more credible environmental information when there are both elaborated self-declared environmental claims and environmental labeling cues on product packaging (Ertz et al., 2017). Further research is needed to investigate when and how different types of info may interact with eco-labeling.

→ FR 23. How do different eco-labels interact and how do eco-labels interact with other types of labels and other information?

### Step 5: Action

#### Conceptual Background

If a behavioral intention has been formed, consumers still need to act on it. Not all behavioral intentions are realized. A considerable amount of research investigated the intention-behavior gap (e.g., Pieters and Verplanken, 1995; Davies et al., 2002; Sheeran and Abraham, 2003; Conner and Godin, 2007; Cooke and Sheeran, 2010; Conner et al., 2016) Intentions to consume in a sustainable manner will only be realized if the individual is able to act in the intended manner and perceived benefits for the goal of improving the environment are not outweighed by the perceived costs in terms of other goals. The framework captures the fact that ESFC, like any other goal-directed behavior, always needs to be situated in a broader context that takes into account the full range of abilities and goals of the individual. A first class of hurdles for action relate to the ability of the individual to perform a behavior. If it is impossible or extremely difficult (in reality or as perceived) to perform the intended behavior, it will not take place. For instance, buying an organic food product can be impossible if it is not available or if someone simply does not have the money necessary to buy it. A second reason for not performing an intended behavior relates to other goals that the individual strives for. For instance, it could be that the intended action not only promotes the goal at which it is directed but also hinders the attainment of other goals. If the benefits in terms of one goal are smaller than the costs in terms of other goals, then the intended behavior will not be executed.

Also here, what matters are the subjective beliefs about abilities, costs, and benefits that are often as impactful as objective ones. Sustainable products are often perceived as less aesthetic (Luchs and Kumar, 2017), less performant (Luchs et al., 2010), more effortful (Johnstone and Tan, 2015), and less affordable (Hughner et al., 2007; Gleim et al., 2013). After all, consumers who think they cannot afford organic food products or who think that costs of organic products outweigh the benefits will not buy them. As another example, if consumers eat a specific product primarily because of the joy it brings (e.g., chocolate), they will not be willing to renounce enjoying their regular chocolate by replacing it with insect-based chocolate, unless it brings comparable joy (Lombardi et al., 2019).

#### Interventions to Stimulate Action

##### Ability

###### Decrease (perceived) price

Perceived and actual prices are still a major barrier for ESFC. As a key example, organic food products are generally more expensive than non-organic alternatives (Bezawada and Pauwels, 2013; van Doorn and Verhoef, 2015; Aschemann-Witzel and Zielke, 2017). Held and Haubach (2017) estimate that in the German market, households with a below-median net equivalent income cannot afford to purchase solely organic food products without getting into debt.

Future research could be set up to actually decrease prices of sustainable products or to change price perceptions. In this light, an evolution that offers interesting opportunities for future research, is the growing extent to which food retailers are marketing organic foods as private label foods and the question how organic labeling interacts with (retailer) brand positioning (Jonas and Roosen, 2005; Bauer et al., 2013; Ellison et al., 2016; Konuk, 2018). Here, interdisciplinary research between experts in economics, agriculture, nutrition and psychology would be beneficial to reach a more holistic understanding of the food system and the role of different stakeholders within it.

→ FR 24. Which interventions decrease prices and price perceptions?

The focus of the current review is on high-income countries, but even in these countries, many consumers face financial limitations (Held and Haubach, 2017). Most research (often implicitly) addresses a narrow target group of individuals who are financially able to engage in green consumption. Economic barriers like higher prices and barriers resulting from market imperfections (e.g., limited access to products, lack of information) (Gorynska-Goldmann, 2019) could limit especially the ESFC of less affluent groups. Less affluent consumers may not only have different purchase motives, they also have less access to outlets that offer a wide variety of affordable organic food (Mirsch and Dimitri, 2012). Knowledge on how to get less affluent consumer groups on board is lacking but is a key condition for scaling up sustainable food consumption.

→ FR 25. Which interventions are effective for less affluent target groups?

###### Increase availability

Another barrier to ESFC pertains to perceptions of limited availability of sustainable products (e.g., Feldmann and Hamm, 2015). When it comes to meat consumption, a study showed that almost half of the population (46.3%) considers a vegetarian lifestyle unachievable (Mullee et al., 2017). Specific reasons for not adopting a vegetarian diet included “insufficient vegetarian options” (14.7%), and “insufficient personal cooking skills” (12.3%), although other studies only partially replicated these findings (e.g., Reipurth et al., 2019). Limited accessibility has also been identified as a barrier to buying organic food (Turk and Ercis, 2017).

##### Competing goals

Instead of acting in an environmentally sustainable way to benefit society in the long-term, consumers also want to save money, indulge, or look for a convenient and comfortable way of living in the short-term (Gleim et al., 2013; White and Simpson, 2013; Lanzini and Thøgersen, 2014; Tate et al., 2014). Engaging in ESFC often means setting aside immediate and proximal individual interests for behavior that has consequences for others and are only realized in the future (Spence et al., 2012).

Although people may value the environmental impact of their food choices, at decision time, they can willingly ignore relevant information available to them on the basis of their own feelings toward the object (Gawronski and LeBel, 2008). For instance, consumers can “forget” the environmental impact of red meat because they like eating it.

###### Decrease time pressure

Time pressure could be another barrier of ESFC, even for consumers who report strong environmental concerns (Young et al., 2010). This is especially the case for local food because it may take more time to buy these products (Feldmann and Hamm, 2015). When more automatic processes prevail (for example, when people experience time pressure), consumers are particularly sensitive to both brand information and brand value and are less prone to choose organic/eco brands (Beattie and McGuire, 2016).

###### Provide prompts

Prompts are messages that are given before the behavior occurs to remind the consumer what the desired sustainable behavior is (Lehman and Geller, 2004). Even when individuals feel they have the ability to engage in ESFC, prompts like a sticker on a shopping trolley reminding people to buy seasonal produce, can be a valuable tool to remind motivated people to not forget to act sustainably in line with their sustainability goals. A simple daily text message reminding people of the health or environmental benefits of eating less red meat or processed meat was effective in decreasing consumption (Carfora et al., 2019). Prompts typically contain simple reminders rather than persuasive appeals and work best when people are already motivated to engage in the behavior and for simple behaviors that require very few steps or effort (Gifford, 2011; Osbaldiston and Schott, 2012).

###### Provide feedback

Food choices are often habitual (Neal et al., 2012) in the sense that they occur frequently and automatically in certain contexts (De Houwer, 2019). Berger (2019) proposes to provide immediate digital normative feedback that signals approval about an action at the point of decision making to attempt to break food habits, for example using a “GreenMeter” which graphically displays the cumulative eco-friendliness of food choice immediately after a product is added to the cart. Peloza et al. (2013) also found that reminding people of a time when their behavior was inconsistent with a personally held value leads to subsequent value-consistent behavior. Providing information on how individuals are performing can strengthen people’s beliefs about their capabilities of engaging in a behavior (Bandura, 1997) and has been identified as an effective social influence approach for encouraging environmental behavior (Abrahamse and Steg, 2013), especially when feedback is presented clearly, in real time and over an extended period of time (Chiang et al., 2014). Harkin et al. (2016) show in their meta-analysis that progress monitoring has a robust effect on goal attainment. In this digital era, interactive displays and mobile aps (Flaherty et al., 2018) can become suitable instruments to provide consumers with the information they need at the point of purchase. The interactive nature provides consumers with the control over the information they want to consult while enabling supermarkets to steer consumers by selectively presenting content (van Giesen and Leenheer, 2019). Digital displays with sustainability information increase the time spent in the supermarket and lead to more extensive product comparisons, without necessarily increasing the importance of sustainability cues (van Giesen and Leenheer, 2019). Since consumers are often pressed for time, interactive displays and mobile apps could offer easy and quick access to information in an engaging way. Gamification seems promising as it combines engaging and rewarding aspects of games (Koivisto and Hamari, 2019). Social media could further decrease consumers reluctance to choose eco-products through, for example, user generated content (Kane et al., 2012).

→ FR 26. How can digital displays, mobile apps, gamification and social media trigger ESFC?

###### Facilitate implementation intentions

Forming implementation intentions (i.e., thoughts about what steps to take to engage in action, Gollwitzer, 1999; Kurz et al., 2014) seems a promising tool to increase the probability that behavioral intentions lead to action. Papies (2017) argues that formation of implementation intentions can change the situated conceptualizations that are triggered by situational cues and therefore change behavior. Fennis et al. (2011) showed a positive effect of implementation intentions (e.g., explicitly listing when, where, and how to use a pocket-guide listing sustainable products for a variety of product categories) on sustainable food-purchasing habits. If people identify and imagine a desired future and address potential obstacles with concrete if-then plans that specify when, where, and how to act (a technique called mental contrasting), behavioral intentions clearly translate into actual behavior change (as demonstrated with regard to reduced meat consumption; Loy et al., 2016). Rees et al. (2018) found preliminary evidence that self-monitoring could underlie the effectiveness of implementation intentions (i.e., forming an implementation intention increased the salience of a meat consumption reduction goal).

→ FR 27. How can implementation intentions increase the probability that behavioral intentions are translated in actual ESFC actions?

###### Counteract disgust

Emotional factors are also likely to play a role in acting sustainably and could overshadow environmental goals at the point of purchase. An important barrier to the consumption of cultured meat are feelings of disgust and perceptions of unnaturalness (Verbeke et al., 2015b; Anderson et al., 2019; Circus and Robison, 2019; Shaw and Iomaire, 2019). Also, disgust propensity negatively affects willingness to pay for environmentally sustainable food products like insect-based food products and atypically shaped fruit and vegetables (Powell et al., 2019). Tasting insect-based food can even evoke a state of disgust, reducing taste perceptions (Barsics et al., 2017). Future research could draw from work in developmental psychology that has identified behavioral interventions to counter food neophobia in children (Dovey et al., 2008).

→ FR 28. Which behavioral interventions can counter disgust reactions to environmental sustainable foods that are perceived as (visually) unappealing?

## Further Considerations

The interventions that we put forward in the previous part have all been related to a specific component of the model. However, some interventions sway several components in the goal-directed behavioral process and hence could be deemed more effective (Cadario and Chandon, 2019). Information appeals, for example, pointing out the environmental impact of behavior, can activate personal norms for people who value the environment, can increase environmental values for people who value the environment less or can reassure people and trigger behavioral intentions. As another example, social norm appeals can both increase environmental values, promote ESFC (through the goal of getting approval) and increase perceived discrepancy (through skepticism).

After having reviewed these interventions, we can now formulate some general recommendations for future research investigating interventions that can encourage both short- and long-term ESFC that do not relate to a specific component in the psychological model that we used to structure our review.

First, all efforts need to be part of an integrated approach in order to be optimally effective (Stern, 2000). Berger (2019) found that an approach that combines gamification elements with norm-based feedback (especially feedback based on injunctive norms) effectively steers consumers toward more sustainable food choices. Yokessa and Marette (2019) conclude that it is usually best to combine eco-labeling with other regulatory tools such as standards banning polluting products and including tax mechanisms. On the contrary, Hagmann et al. (2019) suggest that interventions (such as nudges and taxes) can counteract so that people are less willing to support a carbon tax when they get the possibility to be nudged.

→ FR 29. Investigate whether combining different interventions aimed at enhancing ESFC produces add-on effects.

Long-term effects of interventions have not been studied systematically. ESFC will only impact the environment when it is maintained over time (cf. behavior change maintenance in a health context; Conner, 2008; Schwarzer, 2008; Kwasnicka et al., 2016). Earlier studies showed that consumers who consider alternative (in this case local) food purchases develop stronger attitudes, and thus get more interested and search for more information on (local) food (Feldmann and Hamm, 2015). Papies (2017) also argues that interventions can result in learning processes triggered by repeatedly performing a new behavior in a given situation or simply from the intervention being present over the long term. The finding that an initial act triggers subsequent similar acts has also often been attributed to changes in self-perception (Burger and Caldwell, 2003; Cornelissen et al., 2008; Van der Werff et al., 2014), environmental values (Sparks et al., 2010; Prooijen and Sparks, 2014) and self-efficacy (Lanzini and Thøgersen, 2014; Lauren et al., 2016). Initial personal commitment especially enhances subsequent sustainable behavior when commitments are made in writing (Lokhorst et al., 2013) or in public (Baca-Motes et al., 2012).

Testing whether nudging could lead to a long-term behavioral change in food consumption also deserves attention (Gifford et al., 2011; Devezer et al., 2014). Loebnitz et al. (2015) suggest that nudging interventions that increase (perceived) availability, for example, could lead to enhanced consumption in the long-run even when the behavioral intervention is taken away, since increased exposure is likely to increase acceptance of unfamiliar or odd products. Future research can test whether keeping nudging interventions longer in place will lead to long-term behavioral change or whether effectiveness will eventually fade away (cf. two-factor theory of Berlyne, 1970). Future research can also test whether variations of nudging interventions are necessary to optimally affect ESFC in the long-run.

Interventions that stimulate buying environmentally friendly products for non-environmental reasons may also affect self-perceptions (cf. positive cueing, Cornelissen et al., 2008) or can crowd out pro-environmental motivations (Schwartz et al., 2015). However, de Groot and Steg (2009) showed that interventions that play on hedonic goals will only stimulate pro-environmental behavior as long as it is pleasurable to do so. Hence, it will also be important to investigate long-term effects of these types of interventions (Albarracin and Wyer, 2001). This long-term effect could be enhanced by giving people a sense of agency (i.e., allowing people to perceive themselves as the causal agents of behavioral outcomes) which could motivate them further to achieve a sustainable goal (van der Weiden et al., 2013).

Similarly, the long-term effect of informational campaigns is also not straightforward. Do people still give attention to these campaigns once they have seen them a couple of times? When is the knowledge provided in these campaigns deep-rooted enough to have an influence in the long-run? Furthermore, it could be tested whether information on (especially) disgust evoking sustainable options takes some time to assimilate and therefore will especially be effective for changing behavior in the long run (Rozin, 2008; Athey et al., 2015; Barsics et al., 2017). Also for economic incentives, research suggests that people get accustomed to price levels, which would decrease the effectiveness of taxes for unstainable products in the long term. Other types of extrinsic incentives could also backfire in the long run (Deci et al., 1999; Evans et al., 2013; Exley, 2017; De Marchi et al., 2019). It may also be optimal to combine different interventions to engender long-term effects (White et al., 2019).

→ FR 30. Test the long-term effects of interventions and how interventions should be adapted to have long-term effects.

Relatedly, some evidence suggests feedback loops from the last step in our model (i.e., action) to the previous steps (see Figure 1), but these feedback loops need to be investigated in a more structured way, both in terms of their prevalence and strength. The current review focused on the psychological side of the ESFC question. Many researchers in the domain of eco-consumption of food, make assumptions about what is and what is not environmentally sustainable. But oftentimes, this question cannot be answered unambiguously. Take organic food: in a critical review, Rosen (2010) points out that the organic food industry has a large financial stake in convincing consumers that organic food is not just organic (which means it is certified to meet a given set of criteria related to the production of the food), but also healthier, tastier, and better for the environment; the latter is, however, not necessarily and unconditionally true. Organic food has the potential to help solve multiple social, economic and ethical problems, but it comes at a higher financial cost and decreases other industries like genetic engineering or artificial add-in production (Toma et al., 2017), which themselves may offer environmental benefits in some circumstances (Adenle et al., 2020). Objective knowledge on the impact of different eco-strategies is needed (local vs. international; in season and international vs. out of season and local). In sum, a simple good vs. bad dichotomy often does not capture the multifaceted reality about the environmental sustainability of food, and in order to move forward, we need to take into account the complex of interrelated stakeholders that together form the global food system (Magrini et al., 2018).

→ FR 31. How to assess sustainability of a food product and how to clearly communicate this environmental impact to customers?

Some future research ideas can be formulated concerning the measurement of ESFC. Beattie and McGuire (2016) argue that human beings have a “divided self” when it comes to the environment and climate change, and this underlying “dissociation” in attitude (implicit versus explicit) might be critical to their behavior as consumers. Future research could investigate the specific relation between implicit and explicit attitudes and ESFC. Implicit and explicit measures toward sustainable products have often been found to be related (Greenwald et al., 2009), although some studies show no correlation (Beattie and McGuire, 2016). Mixed results exist on the predictive nature of implicit and explicit measures (Songa et al., 2019). For example, implicit (rather than explicit) attitudes have been found to influence the use of color-coded carbon footprint information in choosing products while explicit attitudes were not predictive of behavior (Beattie and McGuire, 2016). On the other hand, Panzone et al. (2016) found that Implicit Association Test scores do not significantly predict sustainability of food baskets. Non-vegetarians and vegetarians differ in terms of their implicit attitudes toward plant-based and meat-based foods (Barnes-Holmes et al., 2010; see also De Houwer and De Bruycker, 2007). At this point, however, the exact nature of this correlation (i.e., known-groups approach) is unclear. However, if future research would establish the causal nature of this relationship, one might hypothesize that positive implicit attitudes toward meat could hinder an individual to translate an explicit intention to consume less meat into actual behavior, especially under conditions of automaticity (see Moors and De Houwer, 2006).

The role of implicit attitudes might be expected to be much stronger in food markets, which are characterized by significant time pressure and automaticity (Verplanken and Aarts, 1999; Wood and Neal, 2009). Conversely, Panzone et al. (2016) suggest that explicit attitudes play a more prominent role than implicit attitudes in predicting aggregate measures of consumer behavior supporting earlier research showing that explicit environmental motives are important drivers of behavior change (Thøgersen, 2013).

→ FR 32. Do implicit attitudes predict other sustainable behaviors than explicit attitudes and how can both types of attitudes be changed using the same or different interventions?

Consumers often overestimate their behavior in self-reports (Armitage and Conner, 2001) and self-reports are often unrelated to actual behavior (Moser, 2016). van Dam and van Trijp (2013) show that consumers’ self-reported importance of sustainability is driven by abstract considerations that may be less predictive of actual buying behavior as compared to more realistic, choice-based measures (which tap into what the authors label “determinance”rather than the more abstract “relevance”). The latter finding also resonates in the results reported by Grunert et al. (2014), where respondents expressed relatively high levels of concern with sustainability issues at an abstract level, but lower levels of concern in the context of concrete food choices.

→ FR 33. How to measure attitudes that more closely align to the more concrete level at which actual food choices are being made by consumers in their daily lives?

## Conclusion

It has been widely documented that food preferences, choices and eating habits are hard to change, and likewise, that a substantial gap between favorable attitudes and actual purchase and consumption of more sustainable food products remains to be bridged. By identifying and underpinning a future research agenda, the present review aimed to contribute to tackling the challenge of convincing people to change their eating habits toward more ESFC. First, assuming that food consumption is deliberately or unintentionally directed at attaining goals, a comprehensive theoretical framework of goal-directed behavior was presented as a stepping stone for the proposed research agenda. Second, a critical review of the literature on mechanisms that underlie and explain ESFC (or the lack thereof) in high-income countries was presented and integrated into the goal-directed framework. The resulting types of interventions range from for instance priming and activating personal norms as means to activating environmentally sustainable values, to the use of prompts, feedback, implementation intentions and the countering of disgust and food neophobia as means to foster the enacting of the intended ESFC. Altogether, this analysis yielded a set of 33 future research questions in the interdisciplinary food domain that deserve to be addressed with the aim of fostering ESFC. It offers both researchers and practitioners a guidance for research to untangle the complexity of food-related decisions and to bridge the attitude-behavior gap in ESFC.

## Author Contributions

IV initiated the project and coordinated the review and writing process. JD formulated the conceptual model in cooperation with IV and BW. IV, BW, AV, HD, MG, AS, and WVL reviewed the literature. IV and BW wrote the first draft of the manuscript. All authors wrote sections of the manuscript and contributed to the manuscript revision, read, and approved the submitted and final version.

## Conflict of Interest

The authors declare that the research was conducted in the absence of any commercial or financial relationships that could be construed as a potential conflict of interest.
