# Corrigendum: Environmentally Sustainable Food Consumption: A Review and Research Agenda From a Goal-Directed Perspective

**DOI:** 10.3389/fpsyg.2020.585387

**Published:** 2020-10-21

**Authors:** Iris Vermeir, Bert Weijters, Jan De Houwer, Maggie Geuens, Hendrik Slabbinck, Adriaan Spruyt, Anneleen Van Kerckhove, Wendy Van Lippevelde, Hans De Steur, Wim Verbeke

**Affiliations:** ^1^BE4LIFE, Department of Economics and Business Administration, Ghent University, Ghent, Belgium; ^2^BE4LIFE, Department of Psychology and Educational Sciences, Ghent University, Ghent, Belgium; ^3^BE4LIFE, Department of Agricultural Economics, Ghent University, Ghent, Belgium

**Keywords:** environmental sustainable consumption, environmental sustainable food, goal-directed, positive value, perceived discrepancy, behavioral intention, goal intention, act

The authors wish to clearly state that the framework for environmentally sustainable food consumption (ESFC) put forward in the original article was based not only on the goal-directed framework of Moors et al. (2017) but also on a more recent extension of this framework by Moors and colleagues (Moors, 2019; Köster et al., 2020). In this extended framework, Moors and colleagues provide a systematic overview of the types of problems that can arise in each of the steps of the decision process as well as types of solutions. The specific contribution of the original article lies in (a) the application of this extended framework to the domain of ESFC as a tool for organizing the literature and (b) highlighting behavioral solutions to promote ESFC. As such, the authors organized the literature in terms of the different steps put forward by Moors and colleagues and extend previous literature by identifying interventions that can help people to take these steps to accomplish ESFC.

Certain expressions in the paper (e.g., “our framework”) might incorrectly give the impression that the authors created the extension of the Moors et al. (2017) framework. The authors want to clearly state that the framework and its extension was the work of Moors and colleagues. When the authors referred to “our framework” or when they wrote in the Author Contribution section that Jan De Houwer “formulated the conceptual model” in cooperation with Iris Vermeir and Bert Weijters, their intention was to refer to the framework as it was applied to ESFC. Hence, the framework put forward in the paper corresponds to the goal-directed framework developed by Moors et al. (2017); Moors (2019) and Köster et al. (2020) as applied to ESFC. The authors did indicate that they were “Following the work of Moors et al. (2017)” (p. 3) and that they “focused on the ideas proposed by Moors et al. (2017) because they provide a uniquely detailed overview of the specific components of goal-directed behavior, that is, the various decision steps that people go through, starting from when they set their goal until they accomplish it” which allowed them “to organize the literature on ESFC in terms of these different steps” (p. 3–4). However, the authors want to communicate clearly that the extension of this framework was also developed by Moors and colleagues prior to the current article. Jan De Houwer followed the extended framework and, together with Iris Vermeir and Bert Weijters, applied it in the context of ESFC.

In line with these considerations, the following amendments are made to the section “A Goal-Directed Framework Applied to ESFC and Interventions to Promote ESFC” of the original article:

In the first paragraph, the sentence starting with “Following the work done by Moors et al. (2017), we propose a model that posits five components” is replaced with “Following the work done by Moors et al. (2017) and Moors (2019), we propose a model that posits five components.”

In the third paragraph, the sentence “This allowed us to organize the literature on ESFC in terms of these different steps.” is replaced with the following sentences: “Moreover, we followed the extension of this framework by Moors and colleagues (Moors, 2019; Köster et al., 2020) in which they provided a systematic overview of the types of problems that can arise in each of the steps of the decision process as well as types of solutions. The specific contribution of the current paper lies in (a) the application of this extended framework to the domain of ESFC as a tool for organizing the literature and (b) highlighting behavioral solutions to promote ESFC. As such, we organize the literature in terms of the different steps put forward by Moors and colleagues and extend previous literature by identifying interventions that can help people to take these steps to accomplish ESFC. When, in the remainder of this paper, we refer to “our framework” or “our conceptual model,” we thus refer to the extended framework of Moors (2019) and Köster et al. (2020) as it is applied to ESFC.”

The following reference has been added to the reference section:

Moors, A. (2019, January 7). *Towards a goal-directed account of weak-willed behavior* [Blog post]. Retrieved from: http://philosophyofbrains.com/2019/01/07/empirically-informed-approaches-to-weakness-of-will-a-brains-blog-roundtable.aspx?fbclid=IwAR1UL5uejWUnUttIuAm4dpV3ghkC4i1Xn4x0xQVlxnLU4jDOu6iREiBwxgI (also available on: doi: 10.23668/psycharchives.3126).

The authors apologize for this error and state that this does not change the scientific conclusions of the article in any way. The original article has been updated.
